# Gastric Mucosal Status and Risk of Gastric Cancer in High‐Risk Individuals: A Population‐Based, Multicenter Prospective Study in China

**DOI:** 10.1002/cam4.70331

**Published:** 2024-10-15

**Authors:** Han Liu, Song Song, Xianyi Geng, Ru Chen, Rongshou Zheng, Wenqiang Wei, Ni Li, Wanqing Chen, Jiansong Ren

**Affiliations:** ^1^ Office of Cancer Screening National Cancer Center/National Clinical Research Center for Cancer/Cancer Hospital, Chinese Academy of Medical Sciences and Peking Union Medical College Beijing China; ^2^ National Central Cancer Registry National Cancer Center/National Clinical Research Center for Cancer/Cancer Hospital, Chinese Academy of Medical Sciences and Peking Union Medical College Beijing China

**Keywords:** gastric cancer, gastric mucosal status, incidence, prospective study

## Abstract

**Objective:**

This study explored the distributions of gastric mucosal status and their links to gastric cancer within urban high‐risk individuals in China.

**Methods:**

From 2014 to 2015, a questionnaire survey was administered among individuals aged 40 to 69 years, residing for over 3 years in seven selected cities across China. Utilizing the questionnaire data, high‐risk individuals for gastric cancer were screened. These identified high‐risk individuals were invited to undergo endoscopy, followed by pathological examinations conducted for those with suspicious lesions. Prior cancer patients and those newly diagnosed with gastric cancer were excluded, and the remaining endoscopic participants were prospectively followed up until December 2021.

**Results:**

The prospective study followed 8911 individuals at high risk of gastric cancer for a median duration of 6.77 years. The incidence density of gastric cancer was 58.55 per 100,000 person‐years. At baseline, the distributions of subjects across normal gastric mucosa, gastritis/ulcer/polyp, atrophic gastritis/intestinal metaplasia, and intraepithelial neoplasia groups were 47.09%, 36.27%, 9.57%, and 7.07%, respectively. Compared with normal gastric mucosa, the hazard ratios (HRs) for gastric cancer in the other groups were 0.89 [95% confidence interval (95%CI): 0.38–2.08], 1.52 (95%CI: 0.50–4.66), and 4.63 (95%CI: 1.98–10.82). When using the 40–54 age group as the reference, the HR for 55–69 age group was 3.49 (95%CI: 1.52–7.98).

**Conclusion:**

Among high‐risk individuals with gastric cancer, the proportions of individuals exhibiting different gastric mucosal status inversely correlated with the severity of mucosal lesions, whereas the risk of gastric cancer progressively escalated with the increasing severity of mucosal lesions and advancing age.

## 1 Introduction

Although the overall incidence of gastric cancer has shown a declining trend over the years, the burden of it remains significant, with 968,350 new cases worldwide [1, 2]. The age‐standardized incidence (ASI) of gastric cancer is notably higher in regions such as East Asia, Eastern Europe, and South America, while it is lower in Northern Europe, North America, and Africa [2].

Various factors contribute to the development of gastric cancer, including *Helicobacter pylori* (Hp) infection, smoking, alcohol consumption, overweight or obesity, and diet [3]. These factors work synergistically, transforming normal gastric mucosa (NGM) through stages of nonatrophic gastritis (NAG), atrophic gastritis (AG), intestinal metaplasia (IM), and intraepithelial neoplasia (IN), ultimately leading to gastric cancer [4].

While numerous studies have examined the association between AG, IM, IN, and gastric cancer, there is a dearth of large‐scale prospective studies that concurrently assess the risk of gastric cancer across multiple stages of gastric mucosal status [5, 6, 7, 8, 9, 10]. Among the few such studies, those conducted in Sweden and the Netherlands stand out as classic examples, yet they focused primarily on low‐risk Western populations [11, 12, 13]. The studies regarding this association reported in China were mostly conducted in rural areas [14, 15].

Therefore, there is a lack of research evidence regarding the risk of gastric cancer across various mucosal statuses in China, especially in urban high‐risk individuals. The current study aims to examine the risk of gastric cancer associated with different levels of gastric mucosal status in high‐risk individuals in China in order to provide more evidence for future prevention and control strategies against gastric cancer.

## 2 Methods

### 2.1 Study Population

This population‐based, multicenter, prospective cohort study was initiated in 2014 and conducted until 2015 at baseline, with follow‐up extending until 2021. It encompassed seven cities spanning five provinces in China: Jinan and Qingdao in Shandong Province; Chongqing Municipality; Ningbo and Quzhou in Zhejiang Province; Xuzhou in Jiangsu Province; and Hefei in Anhui Province.

Individuals aged 40 to 69 years, who had resided in these regions for at least 3 years, were invited to voluntarily participate in the study from 2014 to 2015. Exclusion criteria included severe organ dysfunction, mental disorders, a history of cancer, or ongoing treatment for serious medical or surgical conditions. Those who met the inclusion criteria and agreed to participate in the questionnaire survey were assessed for the risk of gastric cancer.

Participants assessed as high risk of gastric cancer were then invited to voluntarily undergo upper gastrointestinal endoscopy at the designated hospitals (Shandong Cancer Hospital, Qingdao Central Hospital, The Fifth People's Hospital of Chongqing, Chongqing Cancer Hospital, Ningbo No. 2 Hospital, Kecheng District People's Hospital of Quzhou, Xuzhou Cancer Hospital, and Anhui Provincial Cancer Hospital) within the agreed timeframe. In cases where suspicious lesions were detected, biopsy samples were taken for pathological examination.

Participants who were diagnosed with gastric cancer at baseline were excluded from the study, and the remaining participants who underwent endoscopy comprised the follow‐up cohort. Excluding those who were followed up for less than half a year, the remaining participants were included in the final analysis.

The study was approved by the ethics committee of China National Cancer Center/Cancer Hospital, Chinese Academy of Medical Sciences, and Peking Union Medical College (approval number: 16‐171/1250).

### 2.2 Questionnaire Survey and High‐Risk Individual Assessment

The questionnaire interviewers underwent standardized training and conducted face‐to‐face interviews to gather comprehensive data, encompassing participants' basic information, dietary habits, lifestyle patterns, psychological and emotional states, medical history, and family history of gastric cancer [16].

Once reviewed, the collected information was input into a high‐risk individuals assessment system, which was primarily designed with reference to the Harvard Cancer Risk Index and weighted risk factors [body mass index (BMI), dietary habits (high‐salt diet, food texture, etc.), alcohol consumption, history of gastric diseases (AG, gastric ulcer, gastric polyps, residual stomach, IM, IN, etc.), and family history of gastric cancer] for gastric cancer among Chinese adults. These factors were determined by a consensus reached by a multidisciplinary expert group, grounded on recent epidemiological data on gastric cancer in China, for the purpose of identifying and screening high‐risk individuals for gastric cancer.

### 2.3 Endoscopy and Pathological Diagnosis

The medical staff underwent rigorous qualification verification, work background assessment, and comprehensive training and certification before commencing their duties [17]. The designated hospitals were either tertiary oncology hospitals with cancer diagnosis and treatment capabilities, or general hospitals with dedicated oncology departments.

Prior to the examination, participants were administered 5–6 mL of 1% lidocaine or pharyngeal spray anesthesia, allowing for a painless and noninvasive insertion of the endoscope. During the procedure, careful observation was made of the color, smoothness, mucus, peristalsis of the mucosa, and the shape of the inner cavity. Abnormal mucosal manifestations were noted, including hyperemia, hemorrhage, erosion, depression, and plaque [18]. After the routine gastroscopy observation, 20 mL of 0.2% indigo carmine was extracted and sprayed sequentially from the gastric antrum to the stomach body and cardia. Postrinsing reobservation was conducted to identify abnormal deposition of the stain in the affected gastric mucosal areas, often resulting in increased staining and uneven changes in the stained region.

In cases of suspicious lesions, biopsy was performed, collecting ≥ 2 specimens for lesions > 1 cm, ≥ 3 specimens for lesions > 2 cm, and ≥ 4 specimens for lesions > 3 cm. The specimens were fixed in 10% neutral buffered formalin solution for 6–48 h, followed by vertical embedding in paraffin wax. Six to eight tissue surfaces were consecutively cut and placed on the same slide, stained with hematoxylin–eosin, sealed, and then sent for pathological examination. If the pathological diagnosis was difficult to determine, expert consultation would be conducted to arrive at a final conclusion. The diagnostic results encompassed a range of findings, including NGM, NAG, AG, low‐grade IN, high‐grade IN, and gastric cancer.

### 2.4 Follow‐Up Procedure

The follow‐up process was conducted in a passive manner. The participants' ID numbers were matched with cancer registry databases and death surveillance databases to obtain the cases and deaths of gastric cancer. The follow‐up was maintained until the occurrence of gastric cancer, death, or December 31, 2021, whichever event transpired first.

### 2.5 Statistical Analyses

Based on endoscopic findings, subjects were divided into four groups: NGM, gastritis/ulcer/polyp (GUP), AG/IM, and IN. Chi‐square tests or Fisher's exact tests were employed to assess the differences between factors in these groups. The incidence density as of 2021 was calculated, and Poisson regression analysis was performed to obtain the corresponding 95% confidence interval (95%CI).

Using the NGM group as the reference, the Cox proportional hazards model was utilized to determine the hazard ratio (HR) and 95%CI for gastric cancer risk in the other groups. Adjustments were made for potential confounding factors, including city and factors with a *p*‐value less than 0.05 in the difference analysis, and subgroup analyses were conducted based on gender and age. The proportional hazards assumption was verified using the Schoenfeld residual method.

Cumulative incidence curves of gastric cancer were plotted for different mucosal statuses, genders, and age groups, utilizing the survminer package and survival package of R software (R version 4.3.2). Statistical significance was set at a *p*‐value of less than 0.05, and all tests were conducted on a two‐sided basis.

## 3 Results

### 3.1 Study Population

**FIGURE 1:**
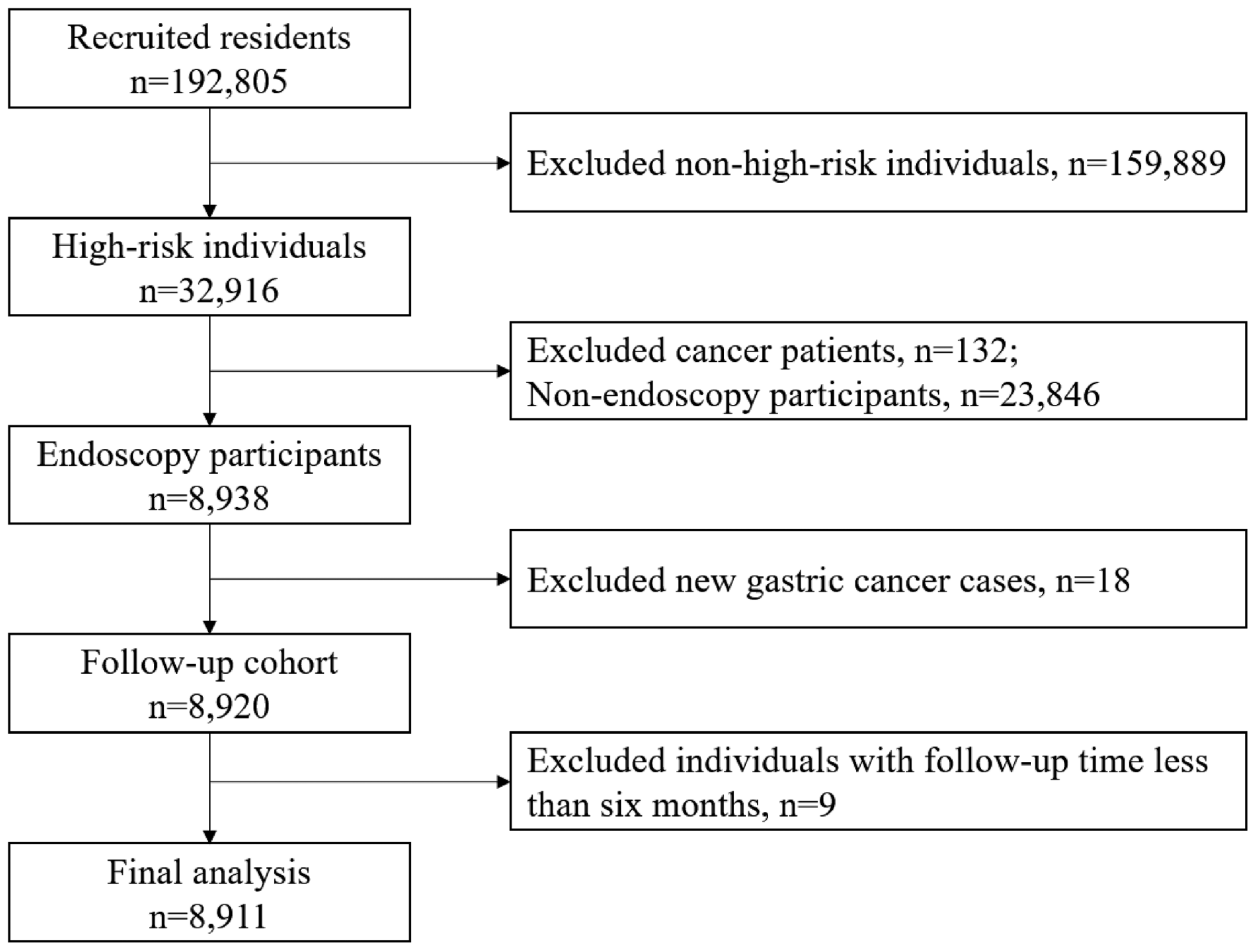
Flowchart of participants included in the study.

Between 2014 and 2015, a total of 192,805 individuals were recruited from seven cities in China. After excluding 159,889 non–high‐risk individuals for gastric cancer, 132 individuals with a history of cancer, 23,846 high‐risk individuals without endoscopy, and 18 individuals with pathological evidence of gastric cancer, the remaining 8920 individuals were included in the follow‐up cohort. Excluding those who were followed up for less than 6 months, the remaining 8911 individuals were included in the final analysis. A flowchart outlining this process is depicted in Figure 1.

### 3.2 Baseline Characteristics

The median age of the participants at baseline was 56.00 years, and 41.97% were males. There were 4196 patients with NGM, 3232 with GUP, 853 with AG/IM, and 630 with IN, and the proportions of them were 47.09%, 36.27%, 9.57%, and 7.07%, respectively, exhibiting a consistent downward trend with escalating severity of mucosal lesions across all subgroups. Significant differences (*p* < 0.05) were observed in the distributions of 17 factors, including age, sex, and BMI, across various populations, with these factors serving as primary adjustment variables for subsequent analyses. The distributions and comparative analysis results of demographic characteristics and potential influencing factors among the groups at baseline are presented in Table 1.

**TABLE 1:** Distribution (%) of gastric mucosal status at baseline (total and by subgroups).

Variable	Total		NGM	GUP	AG/IM	IN	Test	*p*
Total	8911 (100.00)		4196 (47.09)	3232 (36.27)	853 (9.57)	630 (7.07)		
Age							*χ* ^2^ = 47.55	**< 0** **.001**
40–54 years	4140 (100.00)		2096 (50.63)	1447 (34.95)	338 (8.16)	259 (6.26)		
55–69 years	4771 (100.00)		2100 (44.02)	1785 (37.41)	515 (10.79)	371 (7.78)		
Gender							*χ* ^2^ = 94.61	**< 0.001**
Female	5171 (100.00)		2618 (50.63)	1836 (35.51)	428 (8.28)	289 (5.59)		
Male	3740 (100.00)		1578 (42.19)	1396 (37.33)	425 (11.36)	341 (9.12)		
BMI							*χ* ^2^ = 30.12	**< 0.001**
< 24 kg/m^2^	4473 (100.00)		2067 (46.21)	1731 (38.70)	398 (8.90)	277 (6.19)		
≥ 24 kg/m^2^	4438 (100.00)		2129 (47.97)	1501 (33.82)	455 (10.25)	353 (7.95)		
Education							*χ* ^2^ = 11.33	0.079
Primary and below	1833 (100.00)		813 (44.35)	696 (37.97)	189 (10.31)	135 (7.36)		
Junior high school	3358 (100.00)		1570 (46.75)	1217 (36.24)	319 (9.50)	252 (7.50)		
High school and above	3720 (100.00)		1813 (48.74)	1319 (35.46)	345 (9.27)	243 (6.53)		
Marital status							—	0.647
Unmarried	36 (100.00)		21 (58.33)	10 (27.78)	3 (8.33)	2 (5.56)		
Married	8875 (100.00)		4175 (47.04)	3222 (36.30)	850 (9.58)	628 (7.08)		
Vegetable intake							*χ* ^2^ = 1.00	0.802
< 2500 g/week	5339 (100.00)		2514 (47.09)	1952 (36.56)	503 (9.42)	370 (6.93)		
≥ 2500 g/week	3572 (100.00)		1682 (47.09)	1280 (35.83)	350 (9.80)	260 (7.28)		
Fruit intake							*χ* ^2^ = 8.22	**0.042**
< 1250 g/week	6816 (100.00)		3190 (46.80)	2481 (36.40)	637 (9.35)	508 (7.45)		
≥ 1250 g/week	2095 (100.00)		1006 (48.02)	751 (35.85)	216 (10.31)	122 (5.82)		
Red meat intake							*χ* ^2^ = 14.53	**0.002**
≤ 350 g/week	4389 (100.00)		1989 (45.32)	1668 (38.00)	409 (9.32)	323 (7.36)		
> 350 g/week	4522 (100.00)		2207 (48.81)	1564 (34.59)	444 (9.82)	307 (6.79)		
Whole grain intake							*χ* ^2^ = 4.41	0.221
< 500 g/week	6934 (100.00)		3268 (47.13)	2523 (36.39)	642 (9.26)	501 (7.23)		
≥ 500 g/week	1977 (100.00)		928 (46.94)	709 (35.86)	211 (10.67)	129 (6.53)		
High‐salt diet							*χ* ^2^ = 54.71	**< 0.001**
No	4250 (100.00)		1857 (43.69)	1705 (40.12)	388 (9.13)	300 (7.06)		
Yes	4661 (100.00)		2339 (50.18)	1527 (32.76)	465 (9.98)	330 (7.08)		
High‐fat diet							*χ* ^2^ = 49.65	**< 0.001**
No	4869 (100.00)		2129 (43.73)	1890 (38.82)	483 (9.92)	367 (7.54)		
Yes	4042 (100.00)		2067 (51.14)	1342 (33.20)	370 (9.15)	263 (6.51)		
Preserved food							*χ* ^2^ = 94.41	**< 0.001**
No or occasionally	4732 (100.00)		2012 (42.52)	1913 (40.43)	457 (9.66)	350 (7.40)		
Often	4179 (100.00)		2184 (52.26)	1319 (31.56)	396 (9.48)	280 (6.70)		
Smoking							*χ* ^2^ = 25.94	**< 0.001**
No	5302 (100.00)		2554 (48.17)	1953 (36.84)	461 (8.69)	334 (6.30)		
Yes	3609 (100.00)		1642 (45.50)	1279 (35.44)	392 (10.86)	296 (8.20)		
Drinking							*χ* ^2^ = 5.08	0.166
No	4471 (100.00)		2071 (46.32)	1670 (37.35)	413 (9.24)	317 (7.09)		
Yes	4440 (100.00)		2125 (47.86)	1562 (35.18)	440 (9.91)	313 (7.05)		
Physical exercise							*χ* ^2^ = 61.61	**< 0.001**
No	6034 (100.00)		3013 (49.93)	2057 (34.09)	554 (9.18)	410 (6.79)		
Yes	2877 (100.00)		1183 (41.12)	1175 (40.84)	299 (10.39)	220 (7.65)		
Mental trauma							*χ* ^2^ = 77.76	**< 0.001**
No	5769 (100.00)		2542 (44.06)	2213 (38.36)	545 (9.45)	469 (8.13)		
Yes	3142 (100.00)		1654 (52.64)	1019 (32.43)	308 (9.80)	161 (5.12)		
Mental depression							*χ* ^2^ = 63.26	**< 0.001**
No	5820 (100.00)		2574 (44.23)	2224 (38.21)	562 (9.66)	460 (7.90)		
Yes	3091 (100.00)		1622 (52.47)	1008 (32.61)	291 (9.41)	170 (5.50)		
Upper GI disease history							*χ* ^2^ = 27.66	**< 0.001**
No	982 (100.00)		425 (43.28)	357 (36.35)	104 (10.59)	96 (9.78)		
Other	7310 (100.00)		3442 (47.09)	2670 (36.53)	691 (9.45)	507 (6.94)		
IM/dysplasia	619 (100.00)		329 (53.15)	205 (33.12)	58 (9.37)	27 (4.36)		
Hypertension							*χ* ^2^ = 12.39	**0.006**
No	5959 (100.00)		2801 (47.00)	2206 (37.02)	528 (8.86)	424 (7.12)		
Yes	2952 (100.00)		1395 (47.26)	1026 (34.76)	325 (11.01)	206 (6.98)		
Hyperlipemia							*χ* ^2^ = 31.11	**< 0.001**
No	5797 (100.00)		2631 (45.39)	2173 (37.48)	540 (9.32)	453 (7.81)		
Yes	3114 (100.00)		1565 (50.26)	1059 (34.01)	313 (10.05)	177 (5.68)		
Diabetes							*χ* ^2^ = 34.92	**< 0.001**
No	7835 (100.00)		3636 (46.41)	2918 (37.24)	719 (9.18)	562 (7.17)		
Yes	1076 (100.00)		560 (52.04)	314 (29.18)	134 (12.45)	68 (6.32)		
Family history of GC							*χ* ^2^ = 107.18	**< 0.001**
No	5184 (100.00)		2247 (43.34)	2033 (39.22)	524 (10.11)	380 (7.33)		
Second and above	1192 (100.00)		708 (59.40)	325 (27.27)	93 (7.80)	66 (5.54)		
First degree	2535 (100.00)		1241 (48.95)	874 (34.48)	236 (9.31)	184 (7.26)		

*Note:* The values that are bolded statisticall significance at the *P* < 0.05 level and are utilized as crucial adjustment factors in calculating the adjusted risks associated with gastric cancer.

Abbreviations: *χ* ^2^, Chi‐square test; −, Fisher exact; AG, atrophic gastritis; BMI, body mass index; GC, gastric cancer; GI, gastrointestinal; GUP, gastritis/ulcer/polyp; IM, intestinal metaplasia; IN, intraepithelial neoplasia; NGM, normal gastric mucosa.

### 3.3 Follow‐Up Results and Risk of Gastric Cancer

With a median follow‐up of 6.77 (interquartile range: 6.63, 6.93) years until the end of 2021, 35 new cases of gastric cancer were identified. The incidence density of gastric cancer was 58.55 per 100,000 person‐years, with an ASI of 46.55 per 100,000 person‐years based on Segi's world standard population [19].

Specifically, the incidence density for NGM, GUP, AG/IM, and IN groups were 46.44 (95%CI: 26.97–79.99), 41.03 (95%CI: 21.35–78.87), 70.31 (95%CI: 26.39–187.34), and 215.90 (95%CI: 112.34–414.91) per 100,000 person‐years, respectively. Regarding demographics, the incidence density was 91.94 (95%CI: 61.10–138.36) per 100,000 person‐years for males, 34.52 (95%CI: 19.60–60.78) per 100,000 person‐years for females, 25.19 (95%CI: 12.01–52.84) per 100,000 person‐years for those aged 40–54, and 87.52 (95%CI: 60.43–126.76) per 100,000 person‐years for those aged 55–69.

When NGM served as the reference, the HRs for gastric cancer in the other groups demonstrated a linear increase with the severity of mucosal lesions, with HRs of 0.89 (95%CI: 0.38–2.08), 1.52 (95%CI: 0.50–4.66), and 4.63 (95%CI: 1.98–10.82), respectively. This trend persisted after adjustment, and similar findings were observed across all subgroups. Males had an HR of 2.66 (95%CI: 1.33–5.35) compared to females, although this was not statistically significant after adjustment. When using the 40–54 age group as the reference, the HR for the 55–69 age group was 3.49 (95%CI: 1.52–7.98), indicating an increased risk of gastric cancer with advancing age, which persisted in the adjusted HR analysis.

**FIGURE 2:**
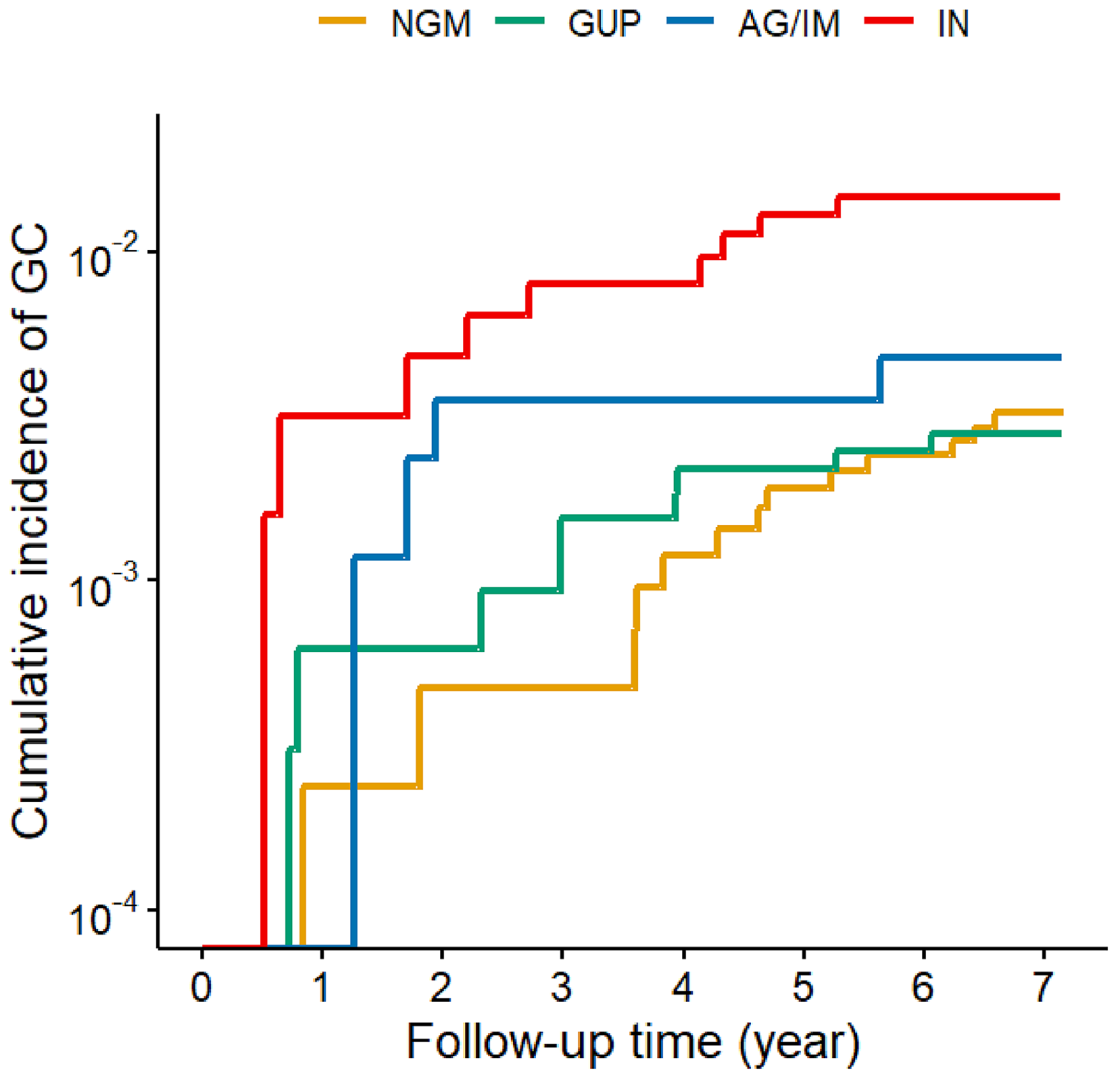
Cumulative incidence of gastric cancer in subjects with different gastric mucosal status at baseline (log‐rank test *p* < 0.05). AG, atrophic gastritis; GC, gastric cancer; GUP, gastritis/ulcer/polyp; IM, intestinal metaplasia; IN, intraepithelial neoplasia; NGM, normal gastric mucosa.

**FIGURE 3:**
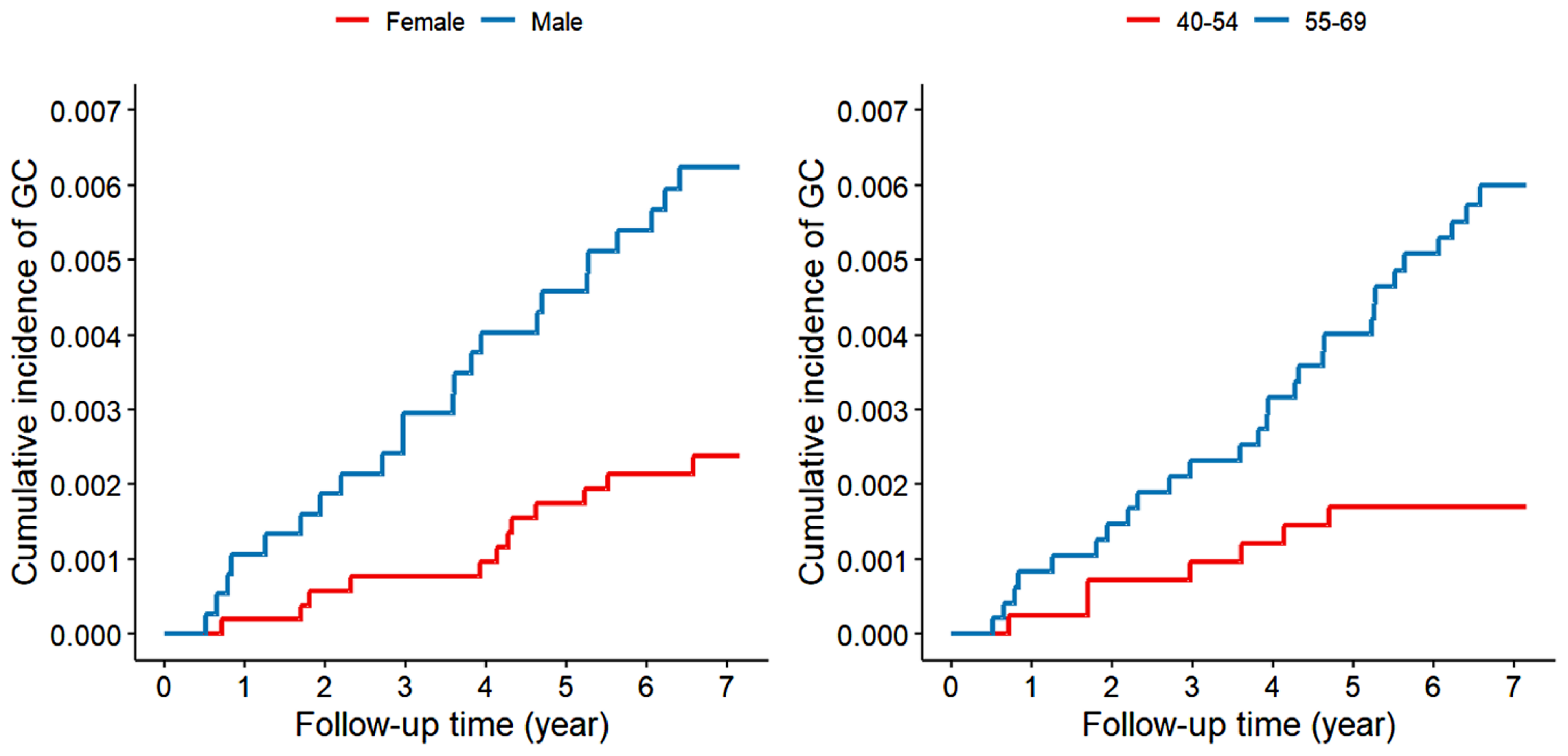
Cumulative incidence of gastric cancer by gender and age groups (log‐rank test *p* < 0.05).

The number of subjects, follow‐up time, number of gastric cancer cases, incidence density, and gastric cancer risk in each group are summarized in Table 2 and Table 3. The cumulative incidence curves, stratified by mucosal status, sex, and age are depicted in Figure 2 and Figure 3.

**TABLE 2:** Risk of gastric cancer in subjects with different gastric mucosal statuses.

	Total *n*	Cases *n*	Follow‐up time year (IQR)	Incidence per 100,000 person‐years (95%CI)	HR (95%CI)	aHR (95%CI)	Trend *p*
Total	8911	35	6.77 (6.63, 6.93)	58.55 (42.04–81.54)	—	—	—
Gastric mucosal status							
NGM	4196	13	6.73 (6.53, 6.90)	46.44 (26.97–79.99)	1	1	0.001
GUP	3232	9	6.87 (6.72, 6.95)	41.03 (21.35–78.87)	0.89 (0.38–2.08)	0.87 (0.30–2.51)	
AG/IM	853	4	6.78 (6.61, 6.95)	70.31 (26.39–187.34)	1.52 (0.50–4.66)	1.31 (0.42–4.11)	
IN	630	9	6.73 (6.66, 6.78)	215.90 (112.34–414.91)	4.63 (1.98–10.82)	5.16 (1.94–13.72)	
Gastric mucosal status (male)							
NGM	1578	7	6.73 (6.54, 6.90)	66.49 (31.70–139.47)	1	1	0.005
GUP	1396	6	6.87 (6.70, 6.95)	63.64 (28.59–141.64)	0.97 (0.33–2.88)	0.85 (0.22–3.23)	
AG/IM	425	3	6.79 (6.61, 6.95)	106.48 (34.34–330.16)	1.62 (0.42–6.25)	1.57 (0.39–6.27)	
IN	341	7	6.72 (6.65, 6.79)	312.25 (148.87–654.96)	4.69 (1.64–13.36)	4.76 (1.46–15.54)	
Gastric mucosal status (female)							
NGM	2618	6	6.73 (6.52, 6.90)	34.36 (15.44–76.48)	1	1	0.364
GUP	1836	3	6.87 (6.72, 6.95)	23.99 (7.74–74.39)	0.69 (0.17–2.78)	0.84 (0.15–4.81)	
AG/IM	428	1	6.77 (6.61, 6.95)	34.83 (4.91–247.24)	1.01 (0.12–8.38)	0.89 (0.10–8.17)	
IN	289	2	6.75 (6.68, 6.78)	103.80 (25.96–415.04)	2.98 (0.60–14.78)	5.82 (0.92–36.91)	
Gastric mucosal status (40–54 years old)							
NGM	2096	2	6.71 (6.51, 6.88)	14.29 (3.57–57.14)	1	1	0.035
GUP	1447	2	6.80 (6.70, 6.94)	20.37 (5.10–81.45)	1.45 (0.20–10.29)	1.57 (0.17–14.78)	
AG/IM	338	1	6.76 (6.58, 6.92)	44.28 (6.24–314.38)	3.11 (0.28–34.29)	2.89 (0.21–40.50)	
IN	259	2	6.73 (6.65, 6.79)	116.42 (29.12–465.50)	8.16 (1.15–57.91)	7.45 (0.76–73.26)	
Gastric mucosal status (55–69 years old)							
NGM	2100	11	6.74 (6.56, 6.91)	78.60 (43.53–141.92)	1	1	0.025
GUP	1785	7	6.89 (6.72, 6.96)	57.78 (27.55–121.19)	0.74 (0.29–1.90)	0.73 (0.21–2.51)	
AG/IM	515	3	6.80 (6.62, 6.95)	87.45 (28.20–271.14)	1.12 (0.31–4.00)	1.08 (0.29–3.99)	
IN	371	7	6.74 (6.67, 6.78)	285.64 (136.18–599.14)	3.61 (1.40–9.30)	5.38 (1.77–16.36)	

Abbreviations: 95%CI, 95% confidence interval; AG, atrophic gastritis; aHR, adjusted hazard ratio, the adjustment factors include city and factors with a *p*‐value less than 0.05 in the baseline difference analysis; GUP, gastritis/ulcer/polyp; HR, hazard ratio; IM, intestinal metaplasia; IN, intraepithelial neoplasia; IQR, interquartile range; NGM, normal gastric mucosa; Trend, linear trend calculated by cox proportional hazards model.

**TABLE 3:** Risk of gastric cancer by gender and age groups.

	Total *n*	Cases *n*	Follow‐up time year (IQR)	Incidence per 100, 000 person‐years (95%CI)	HR (95%CI)	aHR (95%CI)
Total	8911	35	6.77 (6.63, 6.93)	58.55 (42.04–81.54)	—	—
Gender						
Female	5171	12	6.77 (6.63, 6.93)	34.52 (19.60–60.78)	1	1
Male	3740	23	6.77 (6.63, 6.93)	91.94 (61.10–138.36)	2.66 (1.33–5.35)	1.62 (0.58–4.54)
Age						
40–54 years	4140	7	6.75 (6.60, 6.91)	25.19 (12.01–52.84)	1	1
55–69 years	4771	28	6.78 (6.65, 6.94)	87.52 (60.43–126.76)	3.49 (1.52–7.98)	3.46 (1.48–8.11)

Abbreviations: 95%CI, 95% confidence interval; aHR, adjusted hazard ratio, the adjustment factors include city and factors with a *p*‐value less than 0.05 in the baseline difference analysis; HR, hazard ratio; IQR, interquartile range.

## 4 Discussion

We conducted a follow‐up study spanning 6.77 years on 8911 high‐risk individuals for gastric cancer. At baseline, the proportions of subjects across NGM, GUP, AG/IM, and IN groups were 47.09%, 36.27%, 9.57%, and 7.07%, respectively, exhibiting a downward trend with escalating severity of mucosal lesions. Meanwhile, the distributions of factors such as gender, age, diet, lifestyle, psychological status, disease history, and family history of gastric cancer varied among different groups. Similar to previous studies, these factors with different distributions and the city were used as adjustment factors in the subsequent Cox proportional hazards model to calculate the risk of gastric cancer for each mucosal status [20, 21, 22].

Moreover, the incidence density of gastric cancer was 58.55 per 100,000 person‐years, with an ASI of 46.55 per 100,000 person‐years among the 8911 high‐risk individuals. When NGM served as the reference, the HRs for gastric cancer in the other groups demonstrated a linear increase with the severity of mucosal lesions, with HRs of 0.89 (95%CI: 0.38–2.08), 1.52 (95%CI: 0.50–4.66), and 4.63 (95%CI: 1.98–10.82), respectively. When using the 40–54 age group as the reference, the HR for the 55–69 age group was 3.49 (95%CI: 1.52–7.98), indicating an increased risk of gastric cancer with advancing age.

A Swedish study, spanning an average of 10 years and encompassing 288,167 individuals, uncovered 1273 cases of gastric cancer, yielding an incidence density of 53.46 per 100,000 person‐years. Notably, the incidence density and risk of gastric cancer across groups exhibited a consistent, monotonous upward trend, aligning with the findings of this study. However, the study's limitations lay in its exclusive focus on endoscopy timing, gender, age, and pathological outcomes, neglecting to adjust for other potential confounding factors. Moreover, the extended enrollment period from 1979 to 2011, coupled with the absence of standardized guidelines, contributed to significant study heterogeneity [11]. A Chinese study, which followed 14,087 participants for a median of 5.1 years, was a single‐center study in a rural area and also found a trend relationship [15].

This represented the first multicenter, prospective study aimed at elucidating the risk of gastric cancer in individuals with varying mucosal status, with a specific focus on a high‐risk individuals' segment in urban China, providing evidence for future screening and strategic optimization targeting high‐risk individuals for gastric cancer. Adhering to standardized protocols and providing uniform training to researchers, the study was concurrently executed across various locations, featuring a concentrated enrollment phase and an extensive compilation of baseline data. By initially screening the general population for high‐risk individuals and subsequently administering endoscopy, this approach fostered cost‐effectiveness and reduced injury. The detection rate of dysplasia [7.10% (635/8938)] in this study was higher than that in the hospital endoscopy population (6.13%) in Wuxi No. 2 Chinese Medicine Hospital in Jiangsu Province and the local population (2.05%) aged 40–69 in rural areas of Shandong Province, which is a high‐risk area for gastric cancer [23, 24].

Nonetheless, the population grouping in this study was influenced by population size, endoscopic findings, and follow‐up results, and the grouping may not be exhaustive. Follow‐up outcomes did not distinguish cardiac and noncardiac cancers. The adoption of passive follow‐up might overlook cases of gastric cancer and voluntary participation by all subjects underscored the potential influence of participant compliance on the study's results. Additionally, the absence of Hp infection data (due to budget constraints, Hp infection testing was not conducted, and high‐risk population assessment was solely based on the epidemiological information collected through questionnaires) represented a notable gap that could have impacted the findings.

Therefore, in the future, Hp infection detection or other technical methods could be employed to improve the risk assessment effect. Subsequently, special endoscopy could be further utilized for high‐risk individuals to enhance the accuracy of the results [25]. Recognizing the study's relatively brief follow‐up period, which may have precluded sufficient case accumulation for gastric cancer risk estimation, continuous monitoring is advocated. Furthermore, integrating additional surveillance data and active follow‐up strategies could bolster the accuracy of findings. Notably, the study's focus on preselected high‐risk individuals rendered the NGM group's incidence density of 46.44 per 100,000 person‐years, effectively downplaying the comparative risk of other groups.

In conclusion, this population‐based, multicenter prospective study explored the varying proportions of gastric mucosal status, as well as the corresponding risks of gastric cancer stratified by mucosal status, age, and gender within high‐risk individuals. Notably, the proportions of individuals exhibiting different gastric mucosal status inversely correlated with the severity of mucosal lesions, whereas the risk of gastric cancer progressively escalated with the increasing severity of mucosal lesions and advancing age.

## Author Contributions

**Han Liu:** formal analysis (equal), writing – original draft (equal). **Song Song:** formal analysis (equal), writing – original draft (equal). **Xianyi Geng:** writing – original draft (equal). **Ru Chen:** project administration (equal), writing – review and editing (equal). **Rongshou Zheng:** project administration (equal), writing – review and editing (equal). **Wenqiang Wei:** project administration (equal), writing – review and editing (equal). **Ni Li:** project administration (equal), writing – review and editing (equal). **Wanqing Chen:** project administration (equal), writing – review and editing (equal). **Jiansong Ren:** formal analysis (equal), funding acquisition (equal), project administration (equal), writing – review and editing (equal).

## Ethics Statement

Informed consent was obtained in the main manuscript. The study was approved by the ethics committee of China National Cancer Center/Cancer Hospital, Chinese Academy of Medical Sciences, and Peking Union Medical College (approval number: 16‐171/1250).

## Conflicts of Interest

The authors declare no conflicts of interest.

## Data Availability

The data that support the findings of this study are available from the corresponding author upon reasonable request.
